# Visual Hierarchical Processing and Lateralization of Cognitive Functions through Domestic Chicks' Eyes

**DOI:** 10.1371/journal.pone.0084435

**Published:** 2014-01-03

**Authors:** Cinzia Chiandetti, Tommaso Pecchia, Francesco Patt, Giorgio Vallortigara

**Affiliations:** 1 Center for Mind/Brain Sciences, University of Trento, Rovereto (Trento), Italy; 2 Department of Life Sciences - Psychology Unit, University of Trieste, Trieste, Italy; Bowling Green State Universtiy, United States of America

## Abstract

Hierarchical stimuli have proven effective for investigating principles of visual organization in humans. A large body of evidence suggests that the analysis of the global forms precedes the analysis of the local forms in our species. Studies on lateralization also indicate that analytic and holistic encoding strategies are separated between the two hemispheres of the brain. This raises the question of whether precedence effects may reflect the activation of lateralized functions within the brain. Non-human animals have perceptual organization and functional lateralization that are comparable to that of humans. Here we trained the domestic chick in a concurrent discrimination task involving hierarchical stimuli. Then, we evaluated the animals for analytic and holistic encoding strategies in a series of transformational tests by relying on a monocular occlusion technique. A local precedence emerged in both the left and the right hemisphere, adding further evidence in favour of analytic processing in non-human animals.

## Introduction

Every object is defined by features organized in a hierarchical structure: some features occur at a global level and pertain to shape and macroscopic relations, others occur at a local level and are conveyed by fine details and microscopic relations. The determinant feature of an object (at global or local level) may be different with respect to the specific situation the organism is dealing with. The ability to respond to the fundamental feature of an object (relevant dimension or level) is the result of perceptual organization. Navon [1] proposed that perceptual processing proceeds from a global-level analysis to the analysis of local details. This holds for human subjects: when presented with hierarchical stimuli constituted of a large global form made of small local forms, global processing occurs before local analysis is completed, an effect known as global precedence [2].

The global precedence effect represents an ecologically advantageous mechanism that allows an economic use of cognitive resources in the analysis of a scene. In most situations, visual processing stops when the gross features have been processed [3]. It would be reasonable to expect that evolution has maintained the global precedence effect across species and that it may have a long phylogenetic history. Animals can successfully process both levels of hierarchical stimuli, although previous studies have not consistently revealed a global precedence effect (global precedence: cotton-top tamarins [4]; fish [5]; no precedence: chimpanzees [6]; local precedence: baboons [7]; capuchins [8]; macaques [9]; pigeons [10], [11]).

Inconsistent results are likely to depend on procedures and stimuli that differ from study to study, including differences in hemispheric enrolment due to the nature of the tasks. An asymmetric systematization of the functions, with the left hemisphere favouring analytic strategies and the right hemisphere devoted to holistic analysis, is well known in humans and other species [12], [13]. Results collected in a range of neurological and psychiatric patients show that selective lesions to the left hemisphere compromise fine details analysis whereas selective lesions to the right hemisphere result in encoding of the local information only [14].

In a study on pigeons, the animals were trained to categorize complex pictures and tested for hemispheric specialization in the solution of the task. The results revealed that the left hemisphere was mainly involved in conceptualizing the stimulus using category-defining features with an emphasis on the local cues of the image, while the right hemisphere relied more on a memory-based strategy influenced by familiarity of exemplars [15].

However, with respect to hierarchical stimuli, there are no studies investigating a lateralized processing in non-human species. Birds as chicks are especially suitable for the study of cerebral lateralization because of the asymmetrical organization of their visual pathways. Optic nerves, which decussate nearly completely at the optic chiasma, and the absence of central commissures allow the information to be conveyed primarily to the contralateral hemisphere with respect to the eye in use [16]. This could be easily controlled by applying a removable eye-patch [17]. Furthermore, domestic chicks are highly precocial and hence, being tested at an early age, may provide insights into the developmental course of the phenomenon under investigation. Taking advantage of these peculiar characteristics of the chick model, we investigated whether processing of specific aspects of hierarchical stimuli is hemisphere-dependent in a non-human animal.

## Materials and Methods

### Ethics Statement

The study was carried out in compliance with European Community and Italian law on animal experiments by the Ministry of Health, under the authorization of the Ethical Committee of the University of Trieste (protocol number 385 pos II/9 dd 16.03.2012).

### Animals

The experiments were carried out with naïve domestic chicks (*Gallu gallus*) of the Hybro strain (n = 142) hatched in our laboratory from eggs ordered at 7 or 14 days after fertilization and thereafter maintained in darkness. Chicks were reared individually in metal cages (22.5 cm wide × 40 cm long ×30 cm high) lit from above by fluorescent lights (12L:12D) in a room at controlled temperature (30°C). Food was removed 10 hours before starting every training session to obtain the necessary motivational state. Water was always available.

### Apparatus

**Figure 1:**
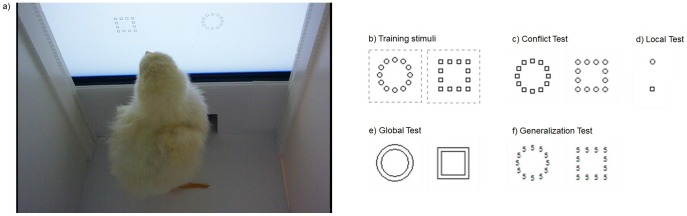
Chick within the apparatus and hierarchical stimuli. A picture of a chick during the training phase in front of the screen while choosing between the two hierarchical stimuli (a). Stimuli used during training (b). Circular configuration of 12 identical circles (left panel) and square configuration of 12 identical squares (right panel) used during training. The dotted lines show the bitmap area for all the stimuli and here shown for the training stimuli for representative purposes only. Stimuli used at Conflict test (c) - the local elements composing the training stimuli are swapped between configurations; Local test (d) - two single local elements of those composing the training stimuli; Global test (e) - two frames covering the global shape of the training stimuli; Generalization test (f) - the local elements composing the training stimuli are replaced with a series of local elements of a new type, a character 5.

Training and testing were conducted in a computer controlled operant chamber (Figure 1a) made of a rectangular box (15.5 cm × 25 cm × 25 cm) with an opening positioned against a 15” TFT monitor provided with an infra-red touch frame. Pecking at the target stimuli automatically exposed a drawer filled with food-grains, otherwise hidden centrally beneath the monitor. Custom software controlled the experimental contingencies and stored them in an output file for the subsequent analysis.

### Procedure

#### Training

The chicks underwent a familiarization phase during their first week after hatching (Day 2-Day 5) and then were trained (Day 8-Day 12) to discriminate between two congruent hierarchical stimuli in a visual concurrent discrimination task. The training stimuli consisted of a circular configuration of 12 indistinguishable circles and a square configuration of 12 indistinguishable squares (Figure 1b). The local elements were drawn in black lines of approximately the same area (circle: 37 pixels; square: 36 pixels). Each stimulus was centred on a white bitmap (70×70 pixels) that served as the target area. The target stimulus was chosen between the two configurations. It was maintained the same for a given chick and it was changed across chicks (n = 73: circular configuration of 12 indistinguishable circles; n = 69: square configuration of 12 indistinguishable squares).

On every training day, a predetermined schedule of corrective (C) and non-corrective (NC) sessions of 20 trials each was administered to the chicks (Table 1). The target and the incorrect stimuli were displayed on the screen at different positions across trials for 60 seconds, balancing left-right target presentation. During C sessions, the chick was able to peck at the screen until the target was correctly chosen, or until 60 seconds had elapsed. An incorrect choice was unrewarded only during a NC training session, by removal of the stimuli from the screen. An inter-trial interval of 3 seconds was established between trials and it was extended to 8 seconds in the case of a correct choice that always resulted in the disappearance of the stimuli. A strict learning criterion was fixed at 85% correct choices in a single NC session on the last training day in order to test only animals that demonstrated an effective target discrimination of either stimuli.

**Table 1:** Schedule.

Training Day	Number of sessions x Type of training	
Day 8	4×C	
Day 9	4×C	
Day 10	1×C	4×NC
Day 11	1×C	4×NC
Day 12	1×C	4×NC

#### Testing

Each chick underwent a single test session that consisted of 20 unrewarded test trials either under monocular (Left Eye-in-use: LE, n = 48; Right Eye-in-use: RE, n = 48) or binocular condition (BIN, n = 46). The monocular occlusions were obtained by applying a removable eye-patch. In these tests, chicks were presented with pairs of novel stimuli which differed from the training stimuli by specific perceptual aspects. We assume that chicks' choices at test revealed the underlying processes used to discriminate between the two original compounds by the hemisphere in use.

In Experiment 1 (n = 54), Conflict test, chicks were asked to choose between two incongruent stimuli obtained by swapping the local elements between the stimuli used for the training (a circular configuration of 12 indistinguishable squares and a square configuration of 12 indistinguishable circles, Figure 1c).

In Experiment 2 (n = 88), chicks were randomly assigned to one of the following test: i) Local test: the choice was between two single local elements of those composing the training stimuli (Figure 1d); ii) Global test: the choice was between two frames covering the global shape of the training stimuli (Figure 1e); iii) Generalization test: the choice was between two hierarchical stimuli obtained by replacing the local elements of the training stimuli with a series of character 5 (Figure 1f).

## Results

### Experiment 1

**Figure 2:**
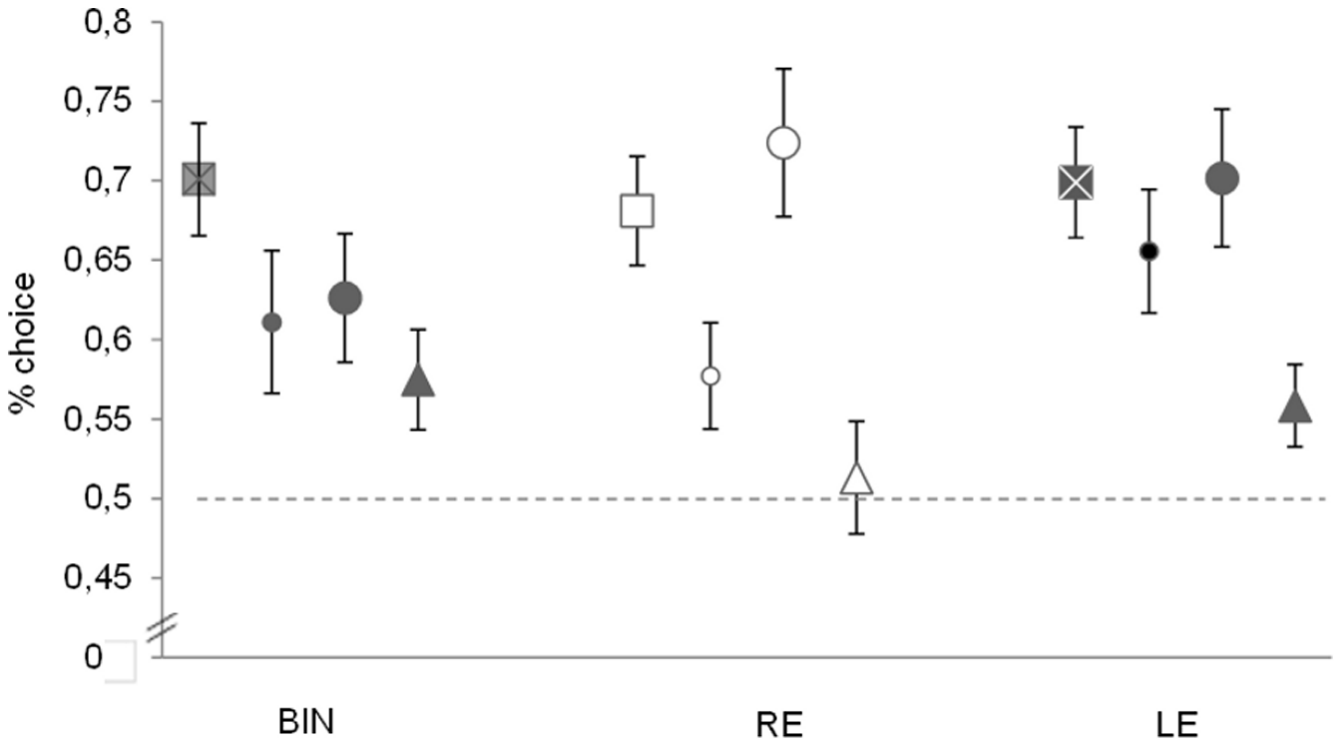
Results. Mean percentage of choice at test (±s.e.m.) is displayed in different symbols (CONFLICT = ▪; LOCAL = 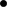; GLOBAL = •; GENERALIZATION = Δ) and colours on the basis of the eye in use (LE  =  grey; RE  =  white; BIN  =  black). In conflict test, the mean represents the choice for the local element coherent with the training stimuli. In the other testing conditions, the means represent a preference for the shape coherent with the training.

Chicks had to choose between two incongruent stimuli, allowing us to investigate whether there was a local or global precedence and whether it was related to the eye in use. A between subject factor analysis of variance was performed with Training Stimulus and Eye in Use as independent factors. The ANOVA revealed that performance at test wasn't affected by the target stimuli used during training (Training Stimulus: F_1,48_ = 1.291, *p* = .261, η_p_^2^ = .026), nor by the eye in use during testing (Eye: F_2,48_ = 0.084, *p* = .920, η_p_^2^ = .003). No interaction between these factors emerged from the analysis (Training Stimulus*Eye: F_2,48_ = 1.643, *p* = .204, η_p_^2^ = .064; Figure 2). Chicks in all visual conditions, and therefore regardless of the hemisphere in use, pecked at the conflict stimuli that presented the same local element of training and they did so significantly more often than expected by chance (LE: t_16_ = 5.6860, *p*<.001; RE: t_16_ = 5.2770, *p*<.001; BIN: t_19_ = 5.7184, *p*<.001, One-sample t-Test).

### Experiment 2

The local precedence effects obtained in the first experiment could be explained either on the basis of a genuine local advantage in processing the hierarchical stimuli or, as an alternative possibility, on the basis of a lack in encoding the global level *tout-court*. Three separate transformational tests were administered to the chicks to check for these alternative explanations. Tests were performed in different visual conditions because differences were expected according to the hemisphere in use.

Three separate ANOVA were performed with Training Stimulus and Test Conditions as independent factors for each eye condition. BIN chicks performed differently across test conditions (F_2,23_ = 5.676, *p* = .01, η_p_^2^ = .33). BIN chicks chose the correct congruent stimuli significantly more often than the incorrect one in all test conditions, although preference for the correct stimulus at the Generalization test was less pronounced when compared to the Local (t_18_ = −2.261, *p* = .036) and Global tests (t_17_ = −3.150, *p* = .006, two-tailed Independent samples t-Test). Chicks were less accurate at the Generalization test, although they were perfectly able to discriminate the stimuli and preferred the congruent one (t_12_ = 2.342, *p* = .037, One-sample t-Test).

LE chicks showed similar performances across test conditions (F_2,28_ = .568, *p* = .573, η_p_^2^ = .04) with a significant preference for the correct congruent stimuli.

RE chicks showed different performances across test conditions (F_2,28_ = 8.176, *p* = .002, η_p_^2^ = .37). No preference emerged for either the correct or the incorrect stimulus in the Generalization test (t_11_ = 0.394, *p* = .701, One-sample t-Test) whereas the correct congruent stimuli were chosen significantly more often in the Local and Global tests. Chicks were more accurate in choosing the correct congruent stimuli in the Global test when compared both to the Local and Generalization tests (Global *vs* Local: t_17_ = 2.788, *p* = .013; Global *vs* Generalization: t_18_ = 3.858, *p* = .001; Local *vs* Generalization: t_21_ = 1.368, *p* = .19, two-tailed Independent samples t-Test).

## Discussion

In Conflict test, chicks preferred the local level in all visual conditions, therefore regardless of the hemisphere in use, adding further evidence in favour of an advantage of analytic perceptual organization in non-human species in similar tasks [7]–[11]. The preference for the local level in this experiment with chicks cannot be explained with a general lack of ability to detect the global level. Indeed, chicks relying on strategies available to either hemispheres or the right hemisphere only showed successful generalization in both the Global and Generalization tests, where the shapes at the local level were respectively removed or replaced.

Chicks relying on strategies prominently available to the left hemisphere failed to recognize a familiar shape from a configural arrangement of novel local elements, confirming that the left hemisphere is more prone to an analytic encoding rather than an analysis of global features [12]. Recognition of the coherent global form at Global test in RE chicks was likely based on local similarities between training and test stimuli. While in the Generalization test the stimulus is still formed by a configuration of novel elements, in the Global test it resembles a single, familiar though bigger element. Hence, RE chicks' choices at Global test may have been determined by the geometric scaling that occurred between training and test stimuli. Indeed, the choice for the enlarged version of the local element may be dictated by the left hemisphere, which processes categorical information, in our case shaped-based, and relies on familiarity. The right hemisphere was still able to perform the Global test. However, the characteristic pattern of choice displayed by right hemisphere was the analysis of the scene on the basis of the configuration of elements composing a global shape. This was not evident for the left hemisphere. The results obtained are in line with the hemispheric differences in categorization found with pigeons in a GO/NOGO paradigm involving pecking behavior [15].

Notably, the right hemisphere showed a local advantage in our experiment, despite its ability to process the global level, as commonly reported in the literature [13]. Since in natural conditions analytic and holistic strategies act in synergy, we argue that in conflict situation the task modulates the involvement of either the left or the right hemispheres and therefore the direction of the advantage (i.e., local or global).

Chicks were tested at two weeks of age and hence, it may be speculated that these results may not extend to adult birds or other vertebrates. Further investigation is needed to check the development of lateralization in adult chickens. However, chicks' attention toward the local level, as demonstrated by the results of the present work, resembles the local precedence displayed by adult vertebrates in comparable tasks [7]–[11]. Furthermore, considering asymmetric behaviours displayed in other tasks by young chicks, no striking differences are anticipated: some forms of visual lateralization are already evident in young individuals (for example, [18]) and comparable to those shown by older subjects of the same and other bird species [19], [20].

Another issue that needs further investigation concerns the effect of incubation condition on the expected lateralized processing of hierarchical stimuli. Here we tested chicks hatched from eggs when the eggs were mainly kept in darkness. It is well established that embryonic light stimulation is a strong environmental trigging factor for some visual asymmetries in birds [21]. In fact, there are lateralized behaviours unaffected by embryonic light exposure and already present in un-stimulated birds [22]. Whether or not light stimulation may change the present pattern of results remains an open question.

An alternative explanation of the results obtained in this work is that the local elements of the stimuli used in the present study, resembling food grains, might have enhanced attention to the local level. On one hand, it is unlikely that two-dimensional circles and squares could be considered *a priori* knowledge representative of food category. Chicks are herbivore and insectivore and therefore feed on a variety of shapes in the wild at all ages. On the other hand, the repetitive association of small circles or squares to food reward with pecking responses may have inescapably concurred to the enhanced local processing. In this view, concurrent discrimination procedures based on pecking behaviour are likely to elicit proximal analysis of the stimuli as in fine discrimination tasks also in the right hemisphere [23]. When the appreciation of the global level is fundamental for the solution of the task, as in navigation, the right hemisphere is in charge favouring global over local analysis [24], [25].
